# Anti-Inflammatory Pharmacotherapy with Ketoprofen Ameliorates Experimental Lymphatic Vascular Insufficiency in Mice

**DOI:** 10.1371/journal.pone.0008380

**Published:** 2009-12-21

**Authors:** Kenta Nakamura, Kavita Radhakrishnan, Yat Man Wong, Stanley G. Rockson

**Affiliations:** Division of Cardiovascular Medicine, Stanford University School of Medicine, Stanford, California, United States of America; Karolinska Institutet, Sweden

## Abstract

**Background:**

Disruption of the lymphatic vasculature causes edema, inflammation, and end-tissue destruction. To assess the therapeutic efficacy of systemic anti-inflammatory therapy in this disease, we examined the impact of a nonsteroidal anti-inflammatory drug (NSAID), ketoprofen, and of a soluble TNF-α receptor (sTNF-R1) upon tumor necrosis factor (TNF)-α activity in a mouse model of acquired lymphedema.

**Methods and Findings:**

Lymphedema was induced by microsurgical ablation of major lymphatic conduits in the murine tail. Untreated control mice with lymphedema developed significant edema and extensive histopathological inflammation compared to sham surgical controls. Short-term ketoprofen treatment reduced tail edema and normalized the histopathology while paradoxically increasing TNF-α gene expression and cytokine levels. Conversely, sTNF-R1 treatment increased tail volume, exacerbated the histopathology, and decreased TNF-α gene expression. Expression of vascular endothelial growth factor-C (VEGF-C), which stimulates lymphangiogenesis, closely correlated with TNF-α expression.

**Conclusions:**

Ketoprofen therapy reduces experimental post-surgical lymphedema, yet direct TNF-α inhibition does not. Reducing inflammation while preserving TNF-α activity appears to optimize the repair response. It is possible that the observed favorable responses, at least in part, are mediated through enhanced VEGF-C signaling.

## Introduction

The lymphatic system is a vascular network that maintains tissue fluid homeostasis and mediates regional inflammatory and immune responses. In addition, the lymphatic system is an important, but often overlooked, participant in cardiovascular physiology and pathology [1]. When the anatomic and functional integrity of the lymphatic vasculature is impaired, the resulting loss of fluid transport capacity leads to lymphedema, the most readily recognizable consequence of lymphatic vascular incompetence [2]. In lymphedema, regional disturbances in lymph transport lead to progressive swelling of dependent tissues and ultimately to permanent, disfiguring changes in tissue architecture.

Acquired lymphedema is a common and disabling state of secondary vascular insufficiency, currently lacking satisfactory pharmacotherapeutics. The potential for therapeutic lymphangiogenesis has been supported by salutary responses to growth factors in animal models of the disease [3]–[8]. However, enthusiasm for the clinical use of these approaches must be tempered by the significant role of cancer in acquired lymphedema [9] and by the potential for enhanced lymph node metastasis [10]–[12].

We have characterized a mouse model of acute, acquired lymphedema [5] that closely simulates the volume response, histopathology, and lymphoscintigraphic characteristics of acquired lymphedema [2], [5], [13]–[14]. This model is a useful experimental platform for elucidating the mechanisms of the disease and for evaluating potential therapies [15]. In previous studies, we showed that administration of vascular endothelial growth factor (VEGF)-C to promote therapeutic lymphangiogenesis reduced edema volume and had beneficial effects on tissue architecture [4], [16]. Molecular characterization of whole-tissue homogenates from mice with lymphedema revealed the prominence of inflammatory mediators such as tumor necrosis factor (TNF-α) [5], underscoring the inflammatory nature of this acquired microvascular disorder. Lymph stasis alone cannot account for the destructive tissue injury associated with lymphedema. The inflammation in lymphedema may be a response to loss of lymphatic vascular integrity and thus a rational target for intervention.

In this study, we investigated the effects of two agents—ketoprofen, a nonsteroidal anti-inflammatory drug (NSAID), and pegsunercept, a modified soluble form of TNF-α receptor R1 (sTNF-R1)—in mice with surgically-induced lymphedema. Ketoprofen reduces inflammation by inhibiting cyclooxygenase but paradoxically increases TNF-α levels [17]–[18]. sTNF-R1 directly inactivates TNF-α and down-regulates TNF-α expression [19]. sTNFR-1 offers advantages over antibody-based inhibitors with regard to administration and dosing while achieving similar effects on TNF-α [20]. Pegylation of the soluble TNF-α receptor increases stability and half-life, addressing a limitation of first-generation soluble receptor inhibitors [21]. Clinically, both classes of antibody- and receptor-based anti-TNF-α drugs have similar therapeutic properties [22]. Our goals were to assess the therapeutic potential of systemic anti-inflammatory therapy and to examine the specific effects of TNF-α modulation in a model of acquired lymphedema.

## Results

### Lymphedema Is Reduced by NSAID and Exacerbated by sTNF-R1

**Figure 1:**
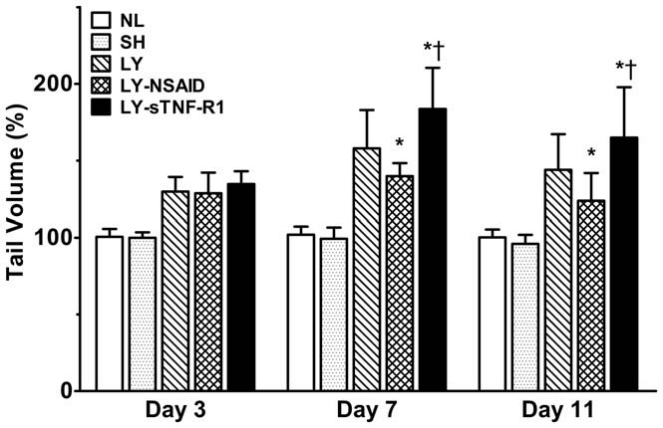
Tail volume response to pharmacotherapy. Changes in tail volume are expressed as a percentage of the volume on day 0. By day 7, lymphedema (LY) mice demonstrate significant increase in tail volume. NSAID-treated lymphedema (LY-NSAID) mice were significantly less edematous than normal controls (NL) or sham surgery control (SH) mice. Conversely, sTNF-R1-treated lymphedema (LY-sTNF-R1) mice were significantly more edematous. *P<0.05 *vs* LY, ^†^ *P*<0.05 *vs* LY-NSAID.

Changes in tail volume are shown in Figure 1. By day 3, tail volume had increased modestly in all three surgical cohorts. On day 7, however, tail volume was 158.0±25.0% of baseline in untreated mice with lymphedema (*P<*5×10^−7^ *vs* sham surgery controls), 139.8±8.5% in NSAID-treated mice (*P*<0.05 *vs* lymphedema), and 183.7±26.8% in sTNF-R1-treated mice (*P*<0.005 *vs* lymphedema). Tail volume changes persisted until sacrifice on day 11. sTNF-R1 had no discernable effect on tail volume in normal and sham surgery mice (data not shown).

### NSAID Therapy Normalizes Histological Changes in Mice with Lymphedema

**Figure 2:**
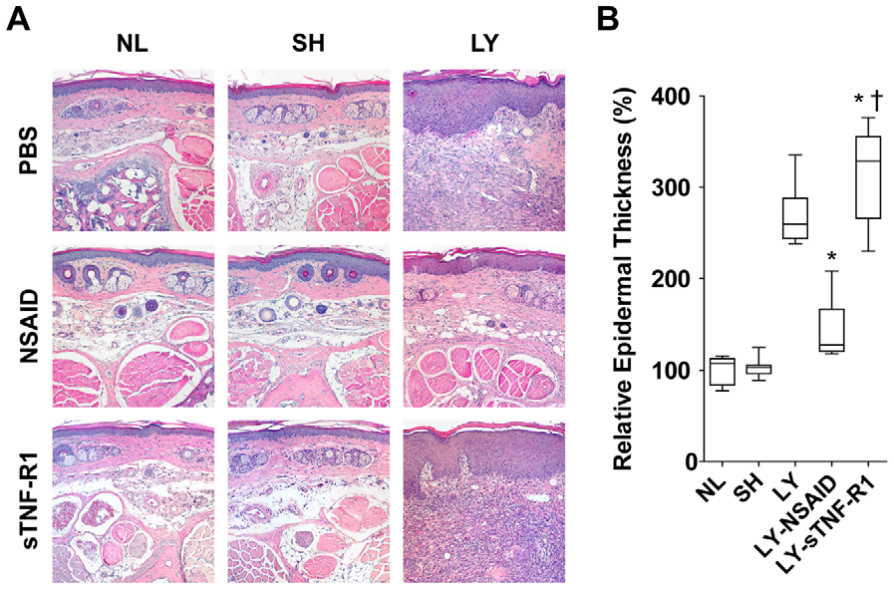
Histological responses to pharmacotherapy. Tail sections were harvested 16 mm from the base of the tail, stained with hematoxylin/eosin, and examined by light microscopy. (**A**) Representative histology. Specimens from normal control (NL) mice, sham surgery control (SH) mice, and lymphedema (LY) mice treated with either PBS, ketoprofen (NSAID), or the TNF-α inhibitor sTNFR-1. Untreated LY show hyperkeratosis, epidermal spongiosis and edema, irregularity of the epidermal/dermal junction, elongation of the dermal papillae, and a 2- to 3-fold expansion of tissue between the bone and the epidermis. There are numerous dilated microlymphatics and increased cellularity, including a large infiltration of neutrophils. Treatment with NSAID normalizes these pathological findings whereas treatment with sTNFR-1 exacerbates the pathology. (**B**) Quantification of epidermal thickness (ET). Changes in ET are expressed as a percentage of the average ET of NL. ET of NSAID-treated lymphedema (LY-NSAID) mice was significantly reduced compared to untreated LY mice (*P*<0.0005) and were not significantly different than NL or SH control mice. ET of sTNF-R1 treated lymphedema (LY-sTNF-R1) mice was significantly increased compared to untreated LY mice (*P*<0.05). *P<0.05 *vs* LY, ^†^ *P*<0.0005 *vs* LY-NSAID.

Qualitative and histomorphometric analysis of paraffin-embedded, hematoxylin/eosin-stained skin sections obtained on day 11 revealed acute inflammatory changes in mice with untreated lymphedema (Figure 2), as we have observed previously [4]–[5]. Normal controls had a thin dermis and epidermis and a normal epidermal/dermal junction. In contrast, mice with untreated lymphedema had hyperkeratosis, epidermal spongiosis and edema, an irregular epidermal/dermal junction, elongated dermal papillae, and a 2- to 3-fold expansion of tissue between the bone and the epidermis. Epidermal thickness of lymphedema mice was 267.0±29.3% of normal control mice (*P*<0.0005). In addition, numerous dilated microlymphatics were evident in the dermis and subdermis, and there was a notable increase in the number of fibroblasts and histiocytes and a large infiltration of neutrophils. There were no histological differences between sham surgery and normal controls.

NSAID therapy resulted in normalization of histological changes, including restoration of normal dermal-epidermal architecture, disappearance of dilated microlymphatics, and marked resolution of inflammatory changes. Epidermal thickness was decreased 47.0±5.1% compared to untreated lymphedema mice (*P*<0.0005) and not significantly different than normal control mice. In contrast, sTNFR1 therapy produced no discernible amelioration; indeed, the epidermal thickness of sTNFR1-treated mice was significantly increased 16.0±2.5% compared to untreated mice with lymphedema (*P*<0.05). Neither therapy affected the histological appearance of post-mortem specimens from normal and sham surgery controls.

### Targeted Gene Expression Analysis by PCR

**Figure 3:**
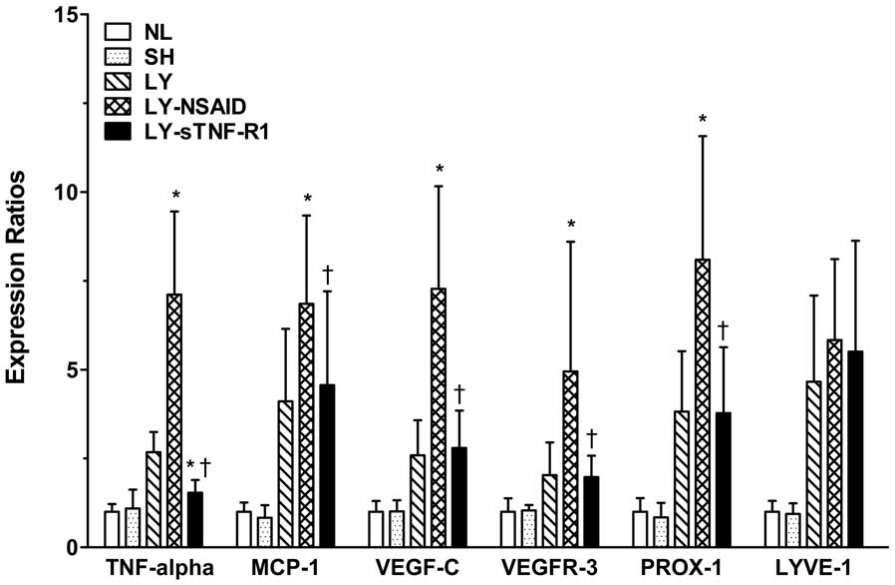
Targeted gene expression analysis by quantitative real-time PCR. Fold-changes of gene expression are relative to normal (NL) controls on day 11. NSAID treatment significantly induced the expression of TNF-α, MCP-1, VEGF-C, VEGFR-3 and Prox1 in lymphedema (LY) mice. The TNF-α inhibitor sTNF-R1 downregulated TNF-α but did not affect other genes. *P<0.05 *vs* LY, ^†^ *P*<0.05 *vs* LY-NSAID.

Differential expression of six targeted, representative genes was quantitated by RT-PCR (Figure 3). Skin was harvested for RNA extraction at the time of sacrifice, on day 11. TNF-α expression was up-regulated in untreated lymphedema. NSAID treatment further increased TNF-α expression (*P*<0.5), while treatment with sTNF-R1 reduced TNF-α expression to control levels (*P*<0.05 *vs* NSAID). A similar pattern of MCP-1 expression was observed. The NSAID-induced up-regulation of these pro-inflammatory cytokines was accompanied by significant, parallel up-regulation of VEGF-C, VEGFR-3, and Prox1 expression. Expression of LYVE-1 did not differ significantly among experimental cohorts.

### Tissue Cytokine Analysis

**Figure 4:**
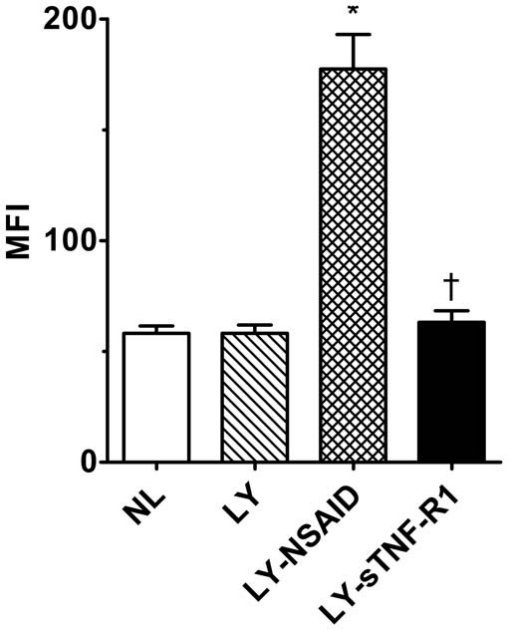
TNF-α levels in tissue homogenates of tail skin. Median fluorescence intensity was used to assess the relative tissue concentrations of TNF-α. TNF-α levels were significantly higher in mice with NSAID-treated lymphedema (LY-NSAID) (*P*<0.005) than in normal controls or mice with untreated or sTNF-R1-treated lymphedema (LY-sTNF-R1). *P<0.005 *vs* untreated lymphedema, ^†^ *P*<0.005 *vs* LY-NSAID.

**Figure 5:**
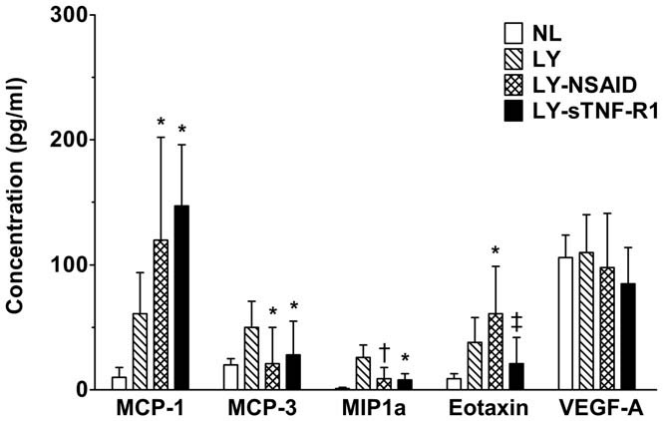
Inflammatory cytokine levels in tissue homogenates of tail skin. Median fluorescence intensity was used to assess the relative tissue concentrations of MCP-1, MCP-3, MIP1a, Eotaxin, and VEGF-A. MCP-1 levels were elevated in mice with lymphedema (LY) and further increased by both ketoprofen and sTNF-R1 anti-inflammatory treatments. MCP-3 levels were also elevated in lymphedema but were significantly reduced by both treatments. A similar pattern was observed for macrophage inflammatory protein 1a (MIP1a). Like TNF-α, eotaxin levels were reduced by NSAID therapy and decreased by sTNF-R1. No effect on the blood vascular growth factor VEGF-A was observed. *P<0.04 *vs* to LY, ^†^ *P* = 0.05 *vs* LY, ^‡^ *P*<0.05 *vs* LY-NSAID.

In view of the disparate effects of the two systemic anti-inflammatory treatments on relative TNF-α gene expression, we quantitated tissue protein levels of this and other cytokines by direct assay of tail skin homogenates (Figures 4 & 5). TNF-α tissue levels were significantly higher in mice with NSAID-treated lymphedema (*P*<0.005) than in normal controls or mice with untreated or sTNF-R1-treated lymphedema. MCP-1 levels, too, were elevated in untreated mice with lymphedema and were further increased by NSAID and sTNF-R1 therapy (*P*<0.04). MCP-3 levels were also elevated in lymphedema, but were significantly reduced by both treatments (*P*<0.04). A similar pattern was observed for macrophage inflammatory protein 1a. Like TNF-α, eotaxin, a potent chemokine for neutrophils, was increased by NSAID therapy (*P*<0.04) and decreased by sTNF-R1 (*P*<0.05). The remaining cytokines assayed (TNF-β; RANTES; interleukins 1a and b, 2–7, 10, and 23; interleukin 12 p40 and p70; interferon-γ; granulocyte and granulocyte-macrophage colony-stimulating factors; IP10; and VEGF-A) showed no significant differences between treated and untreated lymphedema cohorts.

## Discussion

Effective treatment of acquired lymphedema will likely require reversal of both lymphatic vascular insufficiency and inflammation. Our results indicate that systemic attenuation of inflammation and induction of pro-lymphangiogenic factors by the NSAID ketoprofen is an effective therapy for experimental surgically-induced lymphedema.

In the murine tail model, NSAID treatment lessens the profound cellular and molecular inflammatory response that has been described in clinical [2], [14] and experimental lymphedema [5], [13]. We have demonstrated that systemic anti-inflammatory therapy with ketoprofen can ameliorate lymphedema, but the impact upon tissue edema was marginal. Full resolution of the pathology and return to normal microvascular function appear to require more than generic anti-inflammatory intervention alone. Interestingly, the anti-inflammatory effects of ketoprofen were accompanied by robust transcriptional and translational up-regulation of TNF-α, consistent with *in vitro* and *in vivo* studies that demonstrate the direct induction of TNF-α by NSAIDs [17], including ketoprofen [23]–[24]. This relationship is said to constitute a critical role in NSAID-related gastric intolerance [25] and is thought to be mediated by the inhibition of prostaglandin E_2_ [26]–[30], a feedback inhibitor of TNF-α expression. NSAID use is associated with relapse of inflammatory bowel disease [31], further suggesting that up-regulation of TNF-α due to NSAID therapy is clinically relevant.

NSAID treatment significantly increased mRNA and protein levels of TNF-α while other inflammatory mediators such as MCP-3 and MIP1a were inhibited by NSAID treatment in lymphedema mice. This may suggest that NSAID specifically induces TNF-α while exerting otherwise anti-inflammatory effects. MCP-3 is a pro-inflammatory β-chemokine bound by β-Chemokine receptor D6 on a subset of lymphatics [32]. The pattern of D6 expression on lymphatics suggests that it may regulate chemokine-driven trafficking of leukocytes across lymphatics or during lymphangiogenesis [33]. Both NSAID and sTNFR-1 treatment inhibited MCP-3 protein levels, supporting their function as generally anti-inflammatory. The reduction of edema volume after NSAID treatment suggests that anti-inflammatory therapy may also facilitate the normalization of lymphatic vascular function. However, the functional status of the lymphatic vasculature in our model is not directly assessed. We have previous published that MRI can be used to assess the function of lymphatics [34], but the modality remains costly and cumbersome. Functional assessment will be important to further elucidate the mechanism of our observations in future studies.

Across all study categories, the increases in TNF-α mRNA and protein levels correlated with increased expression of the pro-lymphangiogenic factor VEGF-C and its cognate receptor, VEGFR-3. Exogenous administration of VEGF-C lessens the severity and slows the progression of experimental lymphedema, including the murine tail model we used [3]–[4], [16]. TNF-α specifically induces VEGF-C [35]–[39] and Baluk *et al.* recently reported that TNF-α induces lymphangiogenesis in a murine model of airway inflammation [40]. While the effects of TNF-α appear to directly affect the endothelium of the blood vasculature, effects on the lymphatic system may require the inflammatory mediators of recruited leukocytes. The role of the blood vasculature in our model has yet to be explored fully, but the blood vascular growth factor VEGF-A does not appear to be involved in our model. Unraveling the complex interactions between the lymphatic and blood vasculatures and the inflammatory response during lymph stasis will be key to identifying specific targets of therapeutic intervention. Our preliminary studies suggest that modulation of these processes is a viable strategy that may yield clinically relevant therapies.

We found that direct inhibition of TNF-α by sTNF-R1 decreased VEGF-C and VEGFR-3 transcription and expression and correlated with increased disease severity. These findings suggest that TNF-α-mediated up-regulation of VEGF-C expression may be a protective mechanism following lymphatic injury. TNF-α may be required for tissue repair and that it appears to exert a permissive ameliorating effect in our model of acquired lymphedema. We tested this hypothesis by inhibiting TNF-α, which worsened the disease state. Although the mechanisms of VEGF-C-mediated therapy can be debated [41], the positive therapeutic response of augmented VEGF-C signaling can be plausibly linked, at least in part, to enhanced lymphatic repair and increased lymphatic function.

**Figure 6:**
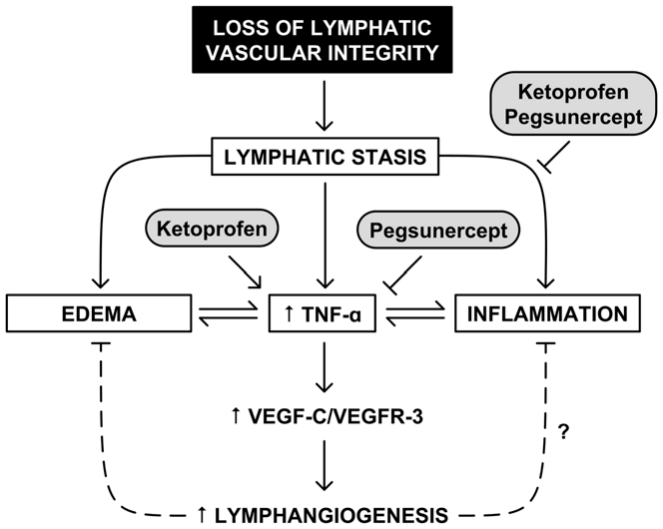
Model of inflammation and lymphedema. Loss of lymphatic vascular integrity leads to diminished lymph transport, which promotes both edema and inflammation. TNF-α, a potent mediator of inflammation, is also a known inducer of the pro-lymphangiogenic factor, VEGF-C. Both ketoprofen and pegsunercept have general inhibitory effects on inflammation, ketoprofen promotes endogenous repair mechanisms mediated by VEGF-C and VEGFR-3by simultaneous inducing TNF-α. In contrast, pegsunercept directly inhibits TNF-α and therefore exacerbates the disease state by disrupting pro-lymphangiogenic processes driven by VEGF-C and VEGFR-3.

Our findings suggest that ketoprofen imparts its beneficial therapeutic response by reducing inflammation and consequent tissue edema. Ketoprofen may also promote VEGF-C-mediated lymphangiogenesis through the induction of TNF-α (Figure 6). This intriguing possibility requires further investigation. The selection of ketoprofen as the agent-of-investigation may have been fortuitous, insofar as the NSAID class of drugs exhibit myriad pharmacological characteristics, with agents of varying IC_50_s and degrees of COX-1, COX-2, and COX-independent mechanisms of action. Extension of our results to other subclasses of NSAIDs will be desirable in future investigations. In addition, whether induction of the VEGF-C/VEGFR-3 axis results in increased lymphatic function, increased *de novo* development of lymphatics, or other alternative mechanisms of benefit is also not yet known and warrants intensive study.

Lymphedema has the distinct morphological attributes of a cutaneous inflammatory disorder [5]. We demonstrated that systemic anti-inflammatory therapy with ketoprofen can ameliorate lymphedema, but the response on end-tissue edema was marginal. Full resolution of the pathology and return to normal microvascular function appear to require more than generic anti-inflammatory intervention alone. We propose that uncoupling the deleterious manifestations of inflammation from the desired pro-lymphangiogenic effects of endogenous repair mechanisms is a logical therapeutic strategy. The present study represents a novel and preliminary step toward the development of pharmacotherapeutics for the treatment of acquired lymphedema.

## Methods

This study was approved by the Administrative Panels on Laboratory Animal Care of Stanford University.

### Creation of Experimental Lymphedema

Tail lymphedema was induced in female hairless, immunocompetent SKH-1 mice (Charles River Laboratories, Boston, MA) as described [5]. The mice were anesthetized with a solution of ketamine, xylazine, and saline (0.07 ml i.p.). The skin of the tail was circumferentially incised 16 mm distal to its base. The major lymphatic trunks were identified by subcutaneous injection of methylene blue distal to the incision and ablated by limited cautery. The mice were carefully monitored for any visceral signs of distress. Sham surgery controls were treated identically, but without lymphatic cautery. Normal controls did not undergo any surgical manipulation. All mice were sacrificed in accordance with American Veterinary Medical Association guidelines for rodent euthanasia after day 11 days of observation. After sacrifice, 500 mg sections of the tail were harvested for paraffin embedding and RNA extraction. For all *in vivo* experiments, housing, husbandry and experimentation were in strict accordance with the Animal Welfare Act and the Guide for the Care and Use of Laboratory Animals.

### Treatment Protocols

Beginning on day 3 after the surgical manipulation, mice with lymphedema received subcutaneous injections of NSAID (ketoprofen, 5 mg/kg; Sigma, St. Louis, MO) (n = 12), sTNF-R1 (pegsunercept, 3 mg/kg; Amgen, Thousand Oaks, CA) (n = 17), or phosphate-buffered saline (PBS) (n = 16) every other day until the day of sacrifice. Sham surgery and normal controls were treated identically with the NSAID (n = 8 per group), sTNF-R1 (n = 11 per group), or PBS (n = 11 normal controls, n = 8 sham surgery controls).

### Tail Volume Quantitation

Tail volume was measured serially in each mouse, using a digital photographic application of the truncated cone approximation [42] as described [43].

### Histology

Immediately after sacrifice, 0.5-g sections of tail were harvested for histological analysis and RNA extraction. Sections extended from a point 4 mm proximal to the surgical incision to 8 mm beyond it. The specimens were fixed overnight in 4% paraformaldehyde, embedded in paraffin, cut into 5-µm sections, stained with hematoxylin and eosin (Richard-Allan Scientific, Kalamazoo, MI), and examined by light microscopy.

### Histomorphometric Quantitation

Epidermal thickness was measured serially using digital images of histology slides stained with hematoxylin and eosin. Processing and analysis were performed using Adobe Photoshop CS4 and ImageJ (http://rsbweb.nih.gov/ij) as previously described with modifications [44]. Quantitative measurements were imported to Prism 5 software (GraphPad Software Inc., La Jolla, CA) for statistical analysis and graphing.

### Quantitative Real-Time RT-PCR

Quantitative real-time polymerase chain reaction (qRT-PCR) was performed as previously described [45]. Primers and probes were from Assays-on-Demand (Applied Biosystems, Foster City, CA). cDNA was synthesized from 5 µg of total RNA with Taqman reverse-transcription reagents (Applied Biosystems) and amplified in triplicate at 50°C for 2 minutes and 95°C for 10 minutes, followed by 40 cycles of 95°C for 15 seconds and 60°C for 1 minute. Reactions without template and/or enzyme were used as negative controls. 18S ribosomal RNA served as an internal control. A standard curve derived from embryonic mouse RNA was plotted for each target gene by linear regression using SPSS software (v. 11.0, Applied Biosystems). RNA quantity was expressed relative to the corresponding 18S control. Fold differences were calculated by dividing the experimental results by the pooled normal results and were plotted on a log_10_ scale.

### Tissue Cytokine Assays

Cytokine levels in tail skin were measured in normal controls and mice with untreated, NSAID-treated, and sTNF-R1-treated lymphedema (n = 3 per group) sacrificed on postoperative day 7. The Luminex LabMap200 System was used to measure the levels of 42 cytokines from the supernatant of tissue homogenates. Multiplex cytokine kits were purchased from Panomics (Fremont, CA), and the assay was performed according to the manufacturer's recommendation with the following modifications: 25–50 µl of samples, standards, and controls were added in duplicate to 96-well filter plates pre-configured with a panel of anti-cytokine antibodies covalently linked to unique polystyrene beads. The plate was incubated overnight at 4°C in the dark with shaking at 500 rpm, vacuum aspirated, and washed three times with 140 µl of wash buffer to remove unbound antigen. Biotinylated detection antibody solution (25 µl) was added to each well and incubated for 1 hour with shaking at 500 rpm at room temperature. The plate was incubated with 50 µl of streptavidin-phycoerythrin for 35 minutes and then with 120 µl of reading buffer for 3 minutes at room temperature with shaking. After laser excitation, digital images of the bead array were captured with a CCD camera and analyzed on a computer workstation with BeadView software.

### Statistical analysis

Two-sample t tests (equal variance) and one-way ANOVA were used to analyze the significance of difference for the tail volume, gene expression, and cytokine assays. *P*<0.05 was considered statistically significant. Standard deviations were calculated for all data.
